# Sleep and Mental Health in Undergraduate Students with Generally Healthy Sleep Habits

**DOI:** 10.1371/journal.pone.0156372

**Published:** 2016-06-09

**Authors:** Helen M. Milojevich, Angela F. Lukowski

**Affiliations:** Department of Psychology and Social Behavior, University of California-Irvine, Irvine, CA, United States of America; University of California, San Francisco, UNITED STATES

## Abstract

Whereas previous research has indicated that sleep problems tend to co-occur with increased mental health issues in university students, relatively little is known about relations between sleep quality and mental health in university students with generally healthy sleep habits. Understanding relations between sleep and mental health in individuals with generally healthy sleep habits is important because (a) student sleep habits tend to worsen over time and (b) even time-limited experience of sleep problems may have significant implications for the onset of mental health problems. In the present research, 69 university students with generally healthy sleep habits completed questionnaires about sleep quality and mental health. Although participants did not report clinically concerning mental health issues as a group, global sleep quality was associated with mental health. Regression analyses revealed that nighttime sleep duration and the frequency of nighttime sleep disruptions were differentially related to total problems and clinically-relevant symptoms of psychological distress. These results indicate that understanding relations between sleep and mental health in university students with generally healthy sleep habits is important not only due to the large number of undergraduates who experience sleep problems and mental health issues over time but also due to the potential to intervene and improve mental health outcomes before they become clinically concerning.

## Introduction

The undergraduate years are a period of vulnerability when considering sleep problems and mental health. Sleep problems tend to worsen over time in undergraduate students [1], a finding which is concerning as even the time-limited experience of significant sleep problems is associated with reduced mental health outcomes [2]. In terms of mental health, approximately 25% of university students who did not report a mental health issue at a baseline assessment ultimately reported a mental health problem 2 years later; approximately 60% of students in that study experienced continuity in the report of at least one mental health issue over time. Approximately 50% of the students who reported continuous mental health issues across assessments did not seek mental health treatment [3]. Taken together, these findings suggest that the sleep problems experienced by undergraduate students may not be inconsequential: they may develop into more significant problems over time and may have implications for mental health outcomes.

Previous research has indicated that sleep problems are associated with poorer mental health outcomes from childhood to adulthood (including increased depression, anxiety, aggression, and delinquent behaviors; see references [4–13]). Existing studies have not yet addressed, however, whether sleep is associated with impairments in mental health in university students with generally healthy sleep habits. Understanding relations between sleep and mental health in this population may be important due to the potential to intervene and improve student mental health outcomes before they become clinically concerning. Global sleep quality is likely an important construct to study in relation to mental health in university students with generally healthy sleep habits as this measure often includes both objective (sleep latency and duration, for example) and subjective elements of sleep (such as enthusiasm for daytime activities and perceived sleep quality) [14]. Addressing the potential differential impact of various components of sleep quality on mental health functioning may also be informative in that global sleep quality and its individual components may be differentially associated with mental health outcomes.

Previous research has established that sleep problems are associated with impaired mental health in childhood and beyond. For example, 2- to 3-year-old children with parent-reported poorer sleep behaviors were more likely to experience increased externalizing (such as inattention-hyperactivity and physical aggression) and internalizing (such as emotional issues and anxiety) problems relative to those with better nighttime sleep habits [4]. Other research suggests that 2- to 5-year-olds with sleep problems are at risk for increased externalizing and total problems, particularly if they obtain fewer than 11 hours of sleep in a 24-hour period [5]. Work conducted with school-aged children replicates and extends these findings. In one report, 7- to 12-year-old children with more fragmented nighttime sleep were rated by their parents as experiencing significantly more delinquent behaviors, thought problems, and total problems relative to children with better sleep habits. Notably, children with more fragmented nighttime sleep received nominally higher scores on each of the included scales, indicating greater problem behaviors pertaining to aggression, attention, withdrawal, somatic complaints, anxiety-depression, and social functioning as well as overall externalizing and internalizing symptomatology [6]. Taken together, these findings indicate that problematic nighttime sleep habits during the school years are related to observable manifestations of behavioral difficulties that may have consequences for child functioning [15–16].

Relations between sleep and mental health have been confirmed and extended in adolescents and young adults. For example, findings from one study indicate that approximately 67% of adolescents with multiple sleep problems (e.g., trouble falling asleep, staying asleep, or early awakening) were classified as having a DSM-relevant anxiety or depressive disorder (see reference [17] for similar work conducted with pre-adolescents). In community samples of adults, poorer sleep quality has been associated with increased reports of externalizing (such as rule-breaking behaviors and physical aggression; see reference [9]) and internalizing (such as depression and anxiety; see references [7–8]) problems relative to what is found in participants with better nighttime sleep quality. Discrete aspects of sleep quality have also been associated with mental health functioning in these samples. In one report, shorter nighttime sleep duration was related to reduced mood and increased anxiety in young adults [10]. Other research suggests that nighttime sleep disturbances, defined as trouble falling asleep, staying asleep, or waking up too early, are associated with both increased anxiety and depression [11–12, 18]. In university students in particular, nighttime sleep disturbances have been shown to predict depression, somatization, obsessive-compulsive behaviors, and psychological distress [13].

Previous research has consistently documented a high prevalence of sleep problems and mental health issues in university students [19–22]. When considering sleep issues, nearly half of approximately 2000 Taiwanese college students reported experiencing sleep problems, with insufficient sleep being the most commonly reported issue [23]. When considering mental health problems, approximately half of American young adults surveyed in a national cross-sectional study reported that they were affected by a clinically-relevant psychiatric problem diagnosed according to DSM-IV criteria within the previous year [24]. Importantly, the majority of those who were diagnosed with an anxiety or mood disorder did not seek any form of treatment, such as visiting a physician or psychologist or taking relevant medications.

As indicated previously, a much larger proportion of university students experiences occasional sleep problems than meets criteria for clinically relevant sleep disorders. Understanding relations between sleep and mental health in individuals with generally healthy sleep habits is important because student sleep habits tend to worsen over time and even time-limited experience of sleep problems may have significant implications for the onset of mental health problems [2]. Findings indicating associations between sleep and mental health in university students with generally healthy sleep habits would suggest that potential benefits of intervening early with the goal of improving mental health outcomes before they become clinically concerning. As such, understanding associations between sleep and mental health in university students with generally healthy sleep habits may have important implications for mental health practices across university campuses.

The goal of the present research was (a) to examine whether self-reported sleep quality and mental health were associated in university students with generally healthy sleep habits. Students were identified as having generally healthy sleep habits if they went to bed before 2:00am at least four nights per week, obtained six or more hours of sleep per night, and scored 10 or less at enrollment on a sleepiness questionnaire. In addition, students with generally healthy sleep habits did not have a history of diagnosed sleep disorders, they did not have abnormal sleep habits, and they did not take nighttime sleep aids more than three nights per week. Because previous research has focused on the differential contributions of nighttime sleep duration and nighttime sleep disruptions to mental health outcomes [10–12, 18, 25–28], an additional goal (b) was to examine whether these aspects of global sleep quality differentially contributed to self-reported mental health. Based on previous research [7–9], we expected that poorer nighttime sleep quality would be related to externalizing and internalizing symptomatology as well as total problems. Finally, we hypothesized that individual variability in nighttime sleep duration and the frequency of nighttime sleep disruptions would differentially predict some of the measured mental health outcomes, particularly total problems and clinically-relevant symptoms of psychological distress.

## Method

### Ethics Statement

As reported previously [29], the completion of this study was approved by the Institutional Review Board at the University of California, Irvine (HS #2010–7686). A waiver of written informed consent was granted for the online screening portion of the study; eligible participants signed informed consent statements before completing the questionnaire battery.

### Participants

Seventy-one students at a large public university in the southwest were selected to participate after having been recruited through the undergraduate participant pool or through advertisements placed on campus. The data from one graduate student were excluded from analysis as well as the data from one undergraduate student who failed to comply with study protocol. The data from 69 undergraduate students (37 females) are presented (mean age = 20 years, range 18 to 25 years). Thirty-four of the participants were of Asian descent, 26 were of Caucasian descent, five were of mixed race, and one was of African American descent; three participants did not report race information. Eleven of the participants identified as Hispanic; one participant did not report ethnicity information. Participants elected to receive either $25 in cash or three points of extra course credit in appreciation for their participation.

### Procedure

#### Recruitment

Participants were selected based on their responses to an online screening questionnaire that was developed for this study based on previous research [30]. The survey included questions about whether respondents were ever diagnosed with a sleep disorder, whether respondents were being treated for a sleep disorder, if they had any abnormal sleep patterns whether they were taking any prescription or over-the-counter sleep aids, on how many nights they went to bed after 2:00am in an average week, how many hours of sleep they obtained on an average night, and if they were taking any psychoactive medications. Participants also completed the Epworth Sleepiness Scale (ESS; [31]) and were asked about their willingness to abstain from caffeine, alcohol, tobacco, and illegal substances during the 12-hour delay between the two study sessions. Finally, potential participants were asked whether English was their native language; students who were not native English speakers were asked to indicate in which language they were most comfortable reading and speaking. These selection criteria were included due to the goals of the larger study from which the presented data were drawn.

Given our interest in examining sleep-mental health associations in university students with generally healthy sleep habits, at least relative to university students recruited more broadly, eligible students went to bed before 2:00am at least four nights per week, obtained six or more hours of sleep per night, and scored 10 or less on the ESS [31]. They also reported that they were native English speakers or were as comfortable conversing and reading in English as they were in their native language. Additionally, eligible students did not have a history of diagnosed sleep disorders, their reported abnormal sleep habits were not identified as abnormal by the researchers (e.g., having trouble sleeping before a major exam), they did not take nighttime sleep aids more than three nights per week, and were not taking any psychoactive medications. Eligible students were also willing to abstain from caffeine, alcohol, tobacco, and illegal substances during their participation in the study. Five hundred fifty-eight students completed the online screening questionnaire but were ineligible to participate: 38 went to bed before 2:00am fewer than three nights per week, 12 commonly obtained less than six hours of sleep per night, 129 scored a 10 or greater on the ESS [31], 11 were not native English speakers or were more comfortable speaking and/or reading in their native language than in English, one was diagnosed with a sleep disorder, three cited abnormal sleep patterns that were unspecified or verified as abnormal by the researchers, six used nighttime sleep aids, five were using psychoactive medications, and 59 were unwilling to adhere to the study protocol; 294 students were ineligible for multiple reasons.

#### Enrollment

Eligible students were contacted by phone or email about continuing their participation in the study. Students who agreed to participate were randomly assigned to arrive at the laboratory for their first session at either 9:00am or 9:00pm, a manipulation that was imposed due to the larger goals of the study from which the presented data were drawn.

#### Questionnaire battery

After informed consent was obtained, each participant completed the questionnaire battery described below. Participants were also provided with contact information for the university counseling center due to the sensitive nature of some items on the questionnaires; participants were instructed to contact them with any mental health issues or concerns.

### Measures

Eligible participants completed a battery of questionnaires that included the ESS [31], the Pittsburgh Sleep Quality Index (PSQI; [14]), and the Adult Self Report Form (ASR; [32]). Participants also completed the Adult Temperament Questionnaire (ATQ; [33]), which is the focus of another report (see reference [29]).

#### Epworth Sleepiness Scale (ESS)

The ESS [31] is an eight-item questionnaire that assesses current sleepiness by asking participants to indicate how likely they would be to doze if they were in particular situations that are differentially soporific, including those in which most people would be expected to fall asleep (e.g., lying down to rest during the afternoon) and others in which only the sleepiest of individuals would be expected to doze (e.g., while sitting and talking with someone). Participants rated each item on a scale from zero (would never doze) to three (high chance of dozing). This measure has high internal consistency and test-retest reliability [34](α = .72 in the current sample). The responses to the 8 items on the ESS [31] were summed to indicate the levels of sleepiness experienced by participants at the start of the first session; higher values are indicative of increased sleepiness.

#### Pittsburgh Sleep Quality Index (PSQI)

The PSQI [14] assesses participant sleep quality over the past 30 days through 19 questions that inquire about quantitative and qualitative aspects of participant sleep habits. Quantitative questions inquire about the time participants usually go to bed at night, the time participants commonly wake up in the morning, the amount of time participants spend asleep at night, and the frequency with which participants experience various nighttime sleep disruptions; qualitative questions inquire about perceived sleep quality and enthusiasm for completing daily activities. This inventory is commonly regarded as the gold standard for assessing subjective sleep quality because it has been validated on individuals with good and poor sleep habits and because it has acceptable levels of internal homogeneity and test-retest stability [14] (α = .61 in the current sample).

Responses to the 19 primary questions on the PSQI [14] were reduced to provide information on seven different components of sleep; these component scores were then summed to yield a global measure of participant sleep quality. Higher composite scores indicate worse sleep quality, with scores greater than five being indicative of categorically poor sleep quality.

We also examined two additional variables from the PSQI in relation to participant mental health. Information about nighttime sleep duration was obtained from one question that inquired about the amount of sleep obtained at night on average over the past month. Information about the frequency of nighttime sleep disruptions was computed from eight questions inquiring as to how often participants experienced trouble sleeping for various reasons. Participant responses to each question were coded from zero (if participants were not affected by the problem) to three (if participants experienced the problem three or more times per week). The codes on these items were then summed to yield a continuous sleep disruption score.

#### Adult Self Report Form (ASR)

Mental health was assessed using the ASR [32]. The ASR asks participants to report on their general behaviors and substance use over the past six months. Participants respond to many of the questions by rating their answers on a scale ranging from zero (not true) to two (very true or often true). Responses to the individual items are combined to yield syndrome scale scores on aggressive behaviors, intrusive problems, and rule-breaking issues (which are combined to yield information on externalizing problems) as well as anxious behaviors, somatic complaints, and withdrawn tendencies (which are combined to yield information on internalizing problems); a total problems score is also obtained. In addition, the ASR yields information on clinically-relevant symptoms of psychological distress including antisocial personality problems, anxiety problems, attention deficit/hyperactivity problems, avoidant personality problems, depressive problems, and somatic problems. Importantly, these scales reflect behaviors that were previously identified as being highly consistent with DSM-IV categories [35]. The ASR has been shown to have high internal consistency and test-retest reliability [32].

The data from the ASR [32] were reduced using the Assessment Data Management (ADM) software available from the publisher. The analyzed output included total scores pertaining to syndrome scales (including internalizing and externalizing disorders) as well as clinically-relevant symptoms of psychological distress (such as anxiety and depression). As described in the ASR manual, the syndrome scales were statistically derived and were comprised of sets of behaviors that tend to co-occur, whereas the DSM-relevant scales were derived from items that a panel of psychiatrists and psychologists deemed consistent with specific DSM-IV diagnostic categories [35]. Although some of the same questions are represented in the scores for the syndrome and the DSM-relevant scales, the latter are more specific and generally include fewer items.

The total scores obtained from the ASR were inspected for outliers, identified as values that were three or more standard deviations above or below the mean. Outlying values were truncated to the highest acceptable value in the dataset plus one, as recommended in reference [36]. The total scores from each scale were *z*-scored before analysis as recommended in the ASR data reduction manual [32].

### Data Analysis

The data were analyzed using SPSS Version 20. The general analytic approach parallels that used to examine associations between participant sleep quality and its components in relation to temperament in this sample [29]. Preliminary analyses of variance (ANOVAs) were conducted to determine whether group assignment, race, and ethnicity were associated with participant sleepiness at the first session, global sleep quality over the past month, and mental health. Correlations were then conducted to examine whether global sleep quality, nighttime sleep duration, and the frequency of nighttime sleep disruptions were associated with the syndrome scales and clinically-relevant symptoms of psychological distress on the ASR. Finally, we conducted multiple regression analyses to examine whether two components of nighttime sleep quality, namely nighttime sleep duration and the frequency of nighttime sleep disruptions, differentially predicted self-reported mental health. We chose to examine these components of nighttime sleep quality as predictors given that they have been related to psychological functioning in previous reports [10–12, 18, 25–28]. Both of these variables were statistically acceptable as candidates for inclusion as separate predictors of mental health, such that they were significantly correlated with sleep quality but were uncorrelated with one another. So as to limit the number of regressions that were conducted, analyses were conducted on the three ASR syndrome scales and six DSM-relevant scales that (a) were correlated with global sleep quality at *p* ≤ .05 and (b) were correlated with either nighttime sleep duration or the frequency of nighttime sleep disruptions at *p* ≤ .10.

## Results

Descriptive statistics for the main study variables, including the percent of participants in the clinical range on each ASR variable, are shown in Table 1.

**Table 1:** Descriptive Statistics for Primary Study Measures (N = 69).

Study Variables	Mean ± SD	Percent in Clinical Range
Epworth Sleepiness Scale		
Sleepiness Score	6.70 ± 3.70	NA
Pittsburgh Sleep Quality Index		
Global Sleep Quality	4.65 ± 2.38	NA
Adult Self Report		
Externalizing Problems	47.29 ± 8.59	0%
Aggressive Problems	51.90 ± 2.93	0%
Intrusive Problems	52.90 ± 5.12	1%
Rule-Breaking Problems	52.71 ± 4.21	0%
Internalizing Problems	50.41 ± 8.79	6%
Anxious Problems	55.00 ± 6.03	3%
Somatic Problems	53.01 ± 5.40	1%
Withdrawn Problems	53.39 ± 5.82	3%
Total Problems	47.99 ± 7.29	0%
DSM-Oriented Scales		
Antisocial Personality Problems	52.87 ± 4.07	0%
Anxiety Problems	54.16 ± 6.24	4%
Attention Deficit/Hyperactivity Problems	53.54 ± 4.70	1%
Avoidant Personality Problems	55.42 ± 6.96	6%
Depressive Problems	53.38 ± 4.37	1%
Somatic Problems	52.54 ± 5.31	1%

Note: Overall scores are reported for the Epworth Sleepiness Scale and the Pittsburgh Sleep Quality Index; T scores are reported for the ASR variables. Borderline clinical scores are not reflected in the percent listed in the clinical range. The presented data are unadjusted for outliers or covariates.

### Preliminary Analyses

Between-subjects analyses of variance (ANOVAs) were conducted to determine whether group assignment (testing at 9:00am or 9:00pm), race (Asian American relative to all other races), and ethnicity (Hispanic relative to non-Hispanic) were associated with participant sleepiness, global sleep quality, and mental health. Participant sex and age were not considered as covariates, as the ASR output is adjusted for these variables. The analyses revealed no differences by group assignment, whereas group differences were found by race and ethnicity on mental health outcomes but not on participant sleepiness or global sleep quality (additional information on specific group differences may be obtained from the second author). Given these significant differences, we included participant race (0 = Asian American or 1 = all other races) and ethnicity (0 = non-Hispanic or 1 = Hispanic) as categorical covariates in all subsequent analyses. As a result, the data from three participants without race values and one participant without an ethnicity value were excluded from the following analyses. Significant results are presented when *p* ≤ .05.

### Bivariate Relations among Sleep Quality and Mental Health

Partial correlations among continuous measures of sleep quality and self-reported mental health are shown in Table 2, whereas partial correlations among the seven PSQI component scores and self-reported mental health are shown in Table 3.

**Table 2:** Correlations among Continuous Sleep Measures and Self-Reported Mental Health.

ASR Factors and Scales	Global Sleep Quality	Nighttime Sleep Duration	Nighttime Sleep Disruptions
Syndrome Scales			
Externalizing Problems	.39*	-.20	.15
Aggressive Problems	.38*	-.22	.02
Intrusive Problems	.03	-.02	.14
Rule-Breaking Problems	.42*	-.16	.18
Internalizing Problems	.35*	-.18	.17
Anxious Problems	.31*	-.21	.06
Somatic Problems	.44*	-.11	.50*
Withdrawn Problems	-.03	-.06	-.20
Total Problems	.46*	-.24	.24
DSM-Oriented Scales			
Antisocial Personality Problems	.37*	-.12*	.07
Anxiety Problems	.41*	-.21	.27*
Attention Deficit/Hyperactivity Problems	.37*	-.18	.20
Avoidant Personality Problems	.12	-.07	-.02
Depressive Problems	.46*	-.23	.20
Somatic Problems	.27*	.00	.55*

Note: Significant findings are indicated (*) when *p* ≤ .05.

**Table 3:** Correlations among PSQI Components (Range 0–3) and Self-Reported Mental Health.

ASR Factors and Scales	Disruptions	Duration	Dysfunction	Efficiency	Latency	Medication	Quality
Syndrome Scales							
Externalizing Problems	.13	.21	.25*	.11	.32*	.10	.33*
Aggressive Problems	-.07	.28*	.25	.08	.34*	.02	.39*
Intrusive Problems	.09	-.02	-.06	-.03	.10	.09	-.04
Rule-Breaking Problems	.27*	.15	.32*	.20	.24	.12	.34*
Internalizing Problems	.07	.19	.15	.39*	.19	-.01	.31*
Anxious Problems	-.05	.22	.18	.35*	.17	-.12	.31*
Somatic Problems	.45*	.19	.02	.36*	.24	.21	.33*
Withdrawn Problems	-.27*	-.02	.00	.13	-.04	.00	.03
Total Problems	.16	.25	.25*	.30*	.29*	.04	.41*
DSM-Oriented Scales							
Antisocial Personality Problems	.06	.22	.20	.11	.32*	.11	.35*
Anxiety Problems	.14	.21	.08	.21	.37*	.01	.44*
Attention-Deficit/Hyperactivity Problems	.21	.17	.32*	.14	.28*	.00	.26*
Avoidant Personality Problems	-.12	.08	.07	.23	.07	-.04	.12
Depressive Problems	.10	.29*	.21	.39*	.30*	-.08	.40*
Somatic Problems	.51*	.02	-.02	.25*	.07	.26*	.18

Note: The continuous sleep efficiency data were truncated to 100% and scored accordingly for 3 participants who reported more time asleep than time spent in bed. Correlations were conducted controlling for participant race (0 = Asian American or 1 = all other races) and ethnicity (0 = non-Hispanic or 1 = Hispanic). Significant findings are indicated (*) when *p* ≤ .05.

### Examining Nighttime Sleep Duration and Disruptions as Predictors of Mental Health

We conducted one regression examining the relation between nighttime sleep duration and the frequency of nighttime sleep disruptions on the syndrome scales pertaining to total problems (Table 4). We then conducted three additional regression analyses on the DSM-relevant scales pertaining to anxiety problems, depressive problems, and somatic complaints (Table 5). Predictors included race and ethnicity (Step 1) and nighttime sleep duration and the frequency of sleep disruptions (Step 2).

**Table 4:** Regressions among Continuous Sleep Measures and Total Problems on the ASR.

	b	SE(b)
Model 1		
Race	-.26	.26
Ethnicity	-.32	.38
Model 2		
Race	-.31	.25
Ethnicity	-.32	.36
Nighttime sleep duration	-.23*	.11
Nighttime sleep disruptions	.07*	.04
*R*^2^		.15
Δ*R*^2^		.12*

Note: Regressions were conducted controlling for participant race (0 = Asian American or 1 = all other races) and ethnicity (0 = non-Hispanic or 1 = Hispanic). Δ*R*^2^ reflects the change in *R*^2^ from Model 1 to Model 2. Significant findings are indicated (*) when *p* ≤ .05.

**Table 5:** Regressions among Continuous Sleep Measures and Select DSM-Relevant Factor Scores.

	Anxiety Problems		Depressive Problems		Somatic Complaints	
	b	SE(b)	b	SE(b)	b	SE(b)
Model 1						
Race	.34	.26	-.25	.26	.28	.26
Ethnicity	-.09	.38	.05	.38	.16	.38
Model 2						
Race	.30	.25	-.30	.25	.28	.23
Ethnicity	-.10	.36	.05	.37	.04	.32
Nighttime sleep duration	-.21	.11	-.22	.11	-.03	.10
Nighttime sleep disruptions	.08*	.04	.06	.04	.16*	.03
*R*^2^		.14		.11		.33
Δ*R*^2^		.12*		.10*		.30*

Note: Regressions were conducted controlling for participant race (0 = Asian American or 1 = all other races) and ethnicity (0 = non-Hispanic or 1 = Hispanic). Δ*R*^2^ reflects the change in *R*^2^ from Model 1 to Model 2. Significant findings are indicated (*) when *p* ≤ .05.

#### Syndrome scales

Nighttime sleep duration was negatively associated with total problems and the frequency of nighttime sleep disruptions was positively associated with total problems. Race and ethnicity were unassociated with total problems.

#### DSM-relevant scales

The frequency of nighttime sleep disruptions was positively associated with increased report of anxiety and somatic complaints but not depressive problems; nighttime sleep duration was not associated with reports of anxiety, depressive problems, or somatic complaints. Race and ethnicity were also unassociated with each of these mental health outcomes.

## Discussion

Understanding relations between sleep and mental health in university students with generally healthy sleep habits is important because student sleep habits tend to worsen over time [1] and even time-limited experience of sleep problems may have significant implications for the onset of mental health problems [2]. The primary goal of the present research was to (a) to examine whether participant sleep quality was associated with self-reported mental health in university students with generally healthy sleep habits and (b) to determine whether two components of sleep quality, namely nighttime sleep duration and the frequency of nighttime sleep disruptions, were differentially predictive of mental health outcomes. The results of this study provide insight as to relations between sleep and mental health in a population of individuals that is at risk for sleep problems [23, 37–38] and psychiatric distress [24]. As such, the findings are relevant not only to undergraduate students but also to university counselors and other officials who have an interest in promoting student health.

The conducted analyses indicated that reduced nighttime sleep quality was associated with various aspects of undergraduate mental health. Specifically, poorer global sleep quality was related to increased externalizing problems, particularly when considering scales associated with aggressive and rule-breaking problems (see reference [9]). Poorer sleep quality was also associated with increased internalizing problems, particularly when considering scales measuring anxiety and somatic complaints (see references [8, 18, 25, 38]). In addition, poorer global sleep quality was related to increased total problems and was widely associated with clinically-relevant symptoms of psychological distress, including increased antisocial personality problems, anxiety problems, attention deficit/hyperactivity problems, depressive problems, and somatic complaints. As such, these data indicate that poorer global sleep quality is broadly associated with reduced mental health in university students—even those with generally healthy sleep habits.

Regression analyses revealed that nighttime sleep duration and the frequency of nighttime sleep disruptions were differentially associated with total problems and certain DSM-oriented scales. In particular, the analyses showed that reduced nighttime sleep duration and increased frequency of nighttime sleep disruptions were associated with self-reported total problems. Examination of clinically-relevant symptoms of psychological distress on the DSM-oriented scales indicated that nighttime sleep disruptions were associated with anxiety problems and somatic complaints but not with depressive problems; nighttime sleep duration was unassociated with mental health on each of the analyzed DSM-relevant scales. Taken together, these findings suggest that although nighttime sleep duration and the frequency of nighttime sleep disruptions may be associated with particular aspects of mental health, other specific sleep-related behaviors not analyzed in this report may be influential as well. As evidence of this, we found significant associations between global sleep quality and DSM-relevant depressive problems, but neither nighttime sleep duration nor the frequency of nighttime sleep disruptions were significant predictors in the conducted regression analysis. Future work is needed to determine which particular aspects of poor sleep quality are associated with reduced functioning in these areas.

One of the primary strengths of this research was in our inclusion of the ASR as a measure of participant mental health. This self-report instrument is a clinically-validated measure of mental health that allows for the analysis of both externalizing and internalizing problems as well as more specific behavioral issues, including those directly related to DSM disorders. As mentioned previously, the ASR has sound psychometric properties and has been used extensively in clinical settings [32]. Although only a small minority of participants in our sample reported mental health scores in the clinical range, this work is clinically relevant in that (a) individuals with sub-clinical sleep problems may go on to develop clinically-relevant sleep disorders and (b) the development of such sleep problems is associated with mental health outcomes. For example, a longitudinal study of over 2000 adults revealed that of the 63% of participants who did not have insomnia at baseline, more than 15% went on to report insomnia only 12 months later [39]. In other research, 13% of young adults who did not have insomnia at baseline developed it during the 3½ year follow-up period [11]. Research also suggests that even the time-limited experience of sleep problems is associated with impaired mental health: in one study, adults who previously reported but were not currently experiencing insomnia were more likely to experience mental health issues than individuals who never reported insomnia. Those who had previously reported but were not currently experiencing insomnia had fewer mental health issues relative to those who were experiencing insomnia at the time or who had experienced continued insomnia across two interviews [2].

In light of the demonstrated associations between poor sleep and mental health problems in this and other research, future studies should be conducted to examine how existing sleep interventions impact mental health, both in students with sleep problems and in those with generally healthy sleep habits. There is a growing body of literature examining the efficacy of sleep interventions in college populations, with most interventions focused on psychoeducational sleep programs, mindfulness or relaxation training, and cognitive behavioral therapy [40–44]. Cognitive behavioral therapy in particular has been effective at improving nighttime sleep [45–47] and mental health outcomes in individuals with [48] and without insomnia (for a review, see reference [49]). Such training programs could potentially serve an important preventative role in promoting healthy sleep habits and mental health outcomes in university students.

Given the potential implications of better understanding relations among sleep and mental health outcomes in university students, future research should address some of the limitations of this research and the larger literature more generally. One of the main limitations of this work is the relatively small sample size that resulted from the imposed exclusion criteria. As mentioned, participants routinely went to bed before 2:00am, they commonly slept for at least 6 hours per night, they did not routinely take sleep aids, and they agreed to abstain from caffeine, alcohol, tobacco, and illegal substances during their participation in the study. As such, our sample may not be representative of the general population of university students, who likely experience more severe sleep problems. As evidence of this, only 11% of university students in previous research [37] received a score indicative of good sleep quality on the Sleep Quality Index [50] whereas 73% of students experienced occasional sleep problems. In our study, 71% of participants had good sleep quality scores on the PSQI [14]. Comparisons of these data must be made cautiously because they were obtained using different instruments with unique means of identifying participants with good sleep quality. However, these data suggest that our participants had generally healthy sleep habits, at least relative to university students who were recruited more broadly in other research. Similarly, our results likely do not generalize to adults with the healthiest of sleep habits (due to, for example, our relatively liberal criterion of recruiting participants who routinely obtained at least 6 hours of sleep at night; the National Sleep Foundation recommends 7–9 hours per night for young adults; see reference [51]). Relatedly, the present study was conducted at one large public university with its own unique culture and sociodemographic characteristics. For these reasons, caution must be taken when attempting to generalize our findings in the absence of replication at other institutions.

Although future work is needed, the conducted research indicates that poorer sleep quality is associated with reduced mental health in university students with generally healthy sleep habits; regression analyses further revealed the manner in which nighttime sleep duration and the frequency of nighttime sleep disruptions predicts mental health outcomes when controlling for participant race and ethnicity. Although only a minority of the tested students experienced clinically concerning mental health issues, understanding relations between sleep and mental health in university students with generally healthy sleep habits is important because (a) undergraduate students may experience worsening sleep over time [1] in association with (b) increasing mental health problems [3]–although whether sleep problems are the cause or consequence of mental health issues remains to be determined. Future research should examine whether existing sleep interventions might improve sleep and mental health outcomes in university students with generally healthy sleep habits so as to improve mental health outcomes before they become clinically concerning. If successful, such prevention efforts could have significant implications for individual students as well as for university campuses more broadly.

## Supporting Information

S1 Dataset The attached supplemental file includes the data analyzed in the present report. (SAV)
